# A Quart of Warts

**Published:** 1858-06

**Authors:** 


### A QUART OF WARTS.

WE saw a laborer with a quart, more or less, of these ugly excrescences on his hands; they had recently appeared. Not all are got rid of in the same way. Some disappear after paring them down so as not to hurt or bleed, and then rubbing into the top of each, with a piece of soft wood, night and morning, two or three drops of oil of vitriol, diluted nitric, or muriatic acid may be used. Others have disappeared after rubbing them with chalk, or a lump of saleratus, moistened, to be persisted in until removed; they sometimes disappear spontaneously.

